# Case report: Cellular therapy for hydroa vacciniforme-like lymphoproliferative disorder in pediatric common variable immunodeficiency with chronic active Epstein-Barr virus infection

**DOI:** 10.3389/fimmu.2022.915986

**Published:** 2022-08-05

**Authors:** Elżbieta Grześk, Sylwia Kołtan, Anna Dąbrowska, Anna Urbańczyk, Jadwiga Małdyk, Bogdan Małkowski, Tomasz Bogiel, Robert Dębski, Krzysztof Czyżewski, Mariusz Wysocki, Jan Styczyński

**Affiliations:** ^1^ Department of Pediatrics, Hematology and Oncology, Faculty of Medicine, Nicolaus Copernicus University in Toruń, Collegium Medicum in Bydgoszcz, Bydgoszcz, Poland; ^2^ Chair and Department of Pathology Medical University of Warsaw, Warsaw, Poland; ^3^ Department of Positron Emission Tomography and Molecular Imaging, Nicolaus Copernicus University in Toruń, Collegium Medicum in Bydgoszcz, Bydgoszcz, Poland; ^4^ Department of Microbiology, Nicolaus Copernicus University in Toruń, Collegium Medicum in Bydgoszcz, Bydgoszcz, Poland

**Keywords:** EBV-CTLs, EBV-specific cytotoxic T cells, CVID - common variable immunodeficiency disorders, Allo-HCT, allogeneic hematopoietic stem cell transplantation, NGS - next generation sequencing, HV-LPD

## Abstract

Hydroa vacciniforme-like lymphoproliferative disorder (HV-LPD) is a cutaneous form of chronic active Epstein-Barrvirus (EBV) infection, which can develop into the extremely rare systemic lymphoma. Patients with Inborn errors of immunity (IEI), such as common variable immunodeficiency (CVID), are at higher risk of developing a severe course of infections especially viral and malignancies than the general population. The aim of the study was to present complex diagnostic and therapeutic management of HV-LPD. The clinical diagnosis was confirmed at the histological and molecular level with next generation sequencing. HV-LPD was diagnosed in a patient with CVID and chronic active Epstein–Barr virus (CAEBV) infection. The patient was refractory to CHOP chemotherapy and immunosuppressive treatment in combination with antiviral drugs (prednisone, bortezomib, gancyclovir). The third-party donor EBV-specific cytotoxic T cells (EBV-CTL, tabelecleucel) were used, which stabilised the disease course. Finally, matched unrelated donor hematopoietic cell transplantation (MUD-HCT) was performed followed by another cycle of EBV-CTL.

## Introduction

Common variable immunodeficiency (CVID) is a primary humoral immunodeficiency, characterized by hypogammaglobulinemia and recurrent, severe infections and increased risk of developing antibody mediated-autoimmune diseases, granulomatous lesions, lymphoid and other types of neoplasms with frequency of 1.5-20.7% in CVID patients (1, 2). The most frequent malignancy is a non-Hodgkin lymphoma (1–3). Additionally, common epithelial tumors of stomach, breast, bladder and cervix can also occur. Pathological mechanisms for development of malignancy in CVID include impaired immune regulation and genetic predisposition. The body’s inability to remove viral and bacterial factors, contributing to the formation of neoplasms, and other iatrogenic causes that increase susceptibility to neoplasia are important as well. Persistent chronic active Epstein-Barr virus (CAEBV) infection is another significant risk factor for lymphoma (4–7).

It is estimated that about 95% of the population aged 20-25 is infected with Epstein-Barr virus (EBV) (3, 7). B cells are classic target cells for EBV, however, T cells, natural killers (NK) and epithelial cells may be infected as well (8).

In immunocompetent individuals, primary infection is usually asymptomatic or in the form of infectious mononucleosis. Subsequently, the infection becomes latent. Its rare form is a chronic active EBV infection (CAEBV), which can manifest itself as fever, lymphadenopathy, splenomegaly, hepatitis, or pancytopenia. Other forms of acute infection, such as hemophagocytic lymphohistiocytosis and chronic EBV infections usually affect people with immunodeficiencies (9–11).

EBV-associated T/NK cell lymphoproliferative diseases (EBV-T/NK-LPD) are a group of heterogeneous and rare diseases resulting from the clonal proliferation of EBV-infected T or NK cells (1, 10, 12). They are more often diagnosed in patients with inborn errors of immunity or secondary immunodeficiency disorders (10, 12–14). Hydroa vacciniforme-like lymphoproliferative disorder (HV-LPD) is one of the very rare forms of EBV-associated diseases (4, 15). Since 2016, HV-LPD has been included in the classification of lymphomas, and since 2018, other forms of lymphomas and mucocutaneous lesions in the course of chronic active EBV infection have been added (16–18). Commonly in HV-LPD cases the hypersensitivity to insect bite was seen (19).

The classic form of HV-LPD with no systemic symptoms or hematological disorders and with high levels of EBV DNA in the blood may be self-limiting. This form is usually diagnosed in patients without documented immunodeficiency. In patients with immune dysfunction, HV-LPD is much more often progressive, with systemic changes, an increased number of T cells with γδTCR in peripheral blood, pancytopenia, lymphadenopathy and organomegaly, uveitis, coronary aneurysms, interstitial pneumonia. Ultimately, it leads to the development of lymphomas (4, 5). In addition, there were also described cases of HV-LPD exacerbation and hemophagocytic lymphohistiocytosis (HLH) (4, 10, 20, 21). Spontaneous elimination of EBV-infected T and NK cells in people with systemic HV-LPD is not possible (10, 17). However, no standard treatment has been established so far. Anti-CD20 monoclonal antibodies are ineffective because they eliminate only EBV-infected B cells. Attempts have been made to remove EBV-infected T or NK cells by the use of immunosuppressants or chemotherapy, but their efficacy was unsatisfactory. Currently, it is assumed that immunosuppressive treatment and/or chemotherapy will reduce the load of EBV-infected T or NK cells, and also minimize the number of EBV copies detected in peripheral blood to <200 IU/ml. The next stage of treatment should be allogeneic hematopoietic cell transplantation (allo-HCT). However, it brings an approximately 10% risk of death due to complications, and a further 10% of patients are likely to have a relapse. An additional problem is the choice of a conditioning regimen administered before HCT, as no clear recommendations have been established so far (9, 10, 22, 23). A solution to the issue of preparing patients with the systemic form of HV-LPD for transplantation, as well as the prevention of recurrence in the early period after HCT, could be the use of immunotherapy with EBV-specific allogeneic cytotoxic T lymphocytes (EBV-CTL) (20). Such attempts have been made with regards to the treatment of EBV-induced post-transplantation lymphoproliferative disorder (PTLD). So far, no information has been found in the available literature to support the validity of this therapeutic concept in the treatment of HV-LPD.

The objective of this report is to present a case of successful treatment of a 14-year-old boy with CVID who was diagnosed with HV-LPD. and successfully treated with immunosuppressants, standard chemotherapy, immunotherapy with EBV-specific allogeneic cytotoxic T cells and HCT. To our knowledge, this is the first clinical description showing the importance of specific immunotherapy in preparation for allo-HCT and in the period of immune reconstruction after transplantation in children.

## Case description

**Figure 1:**
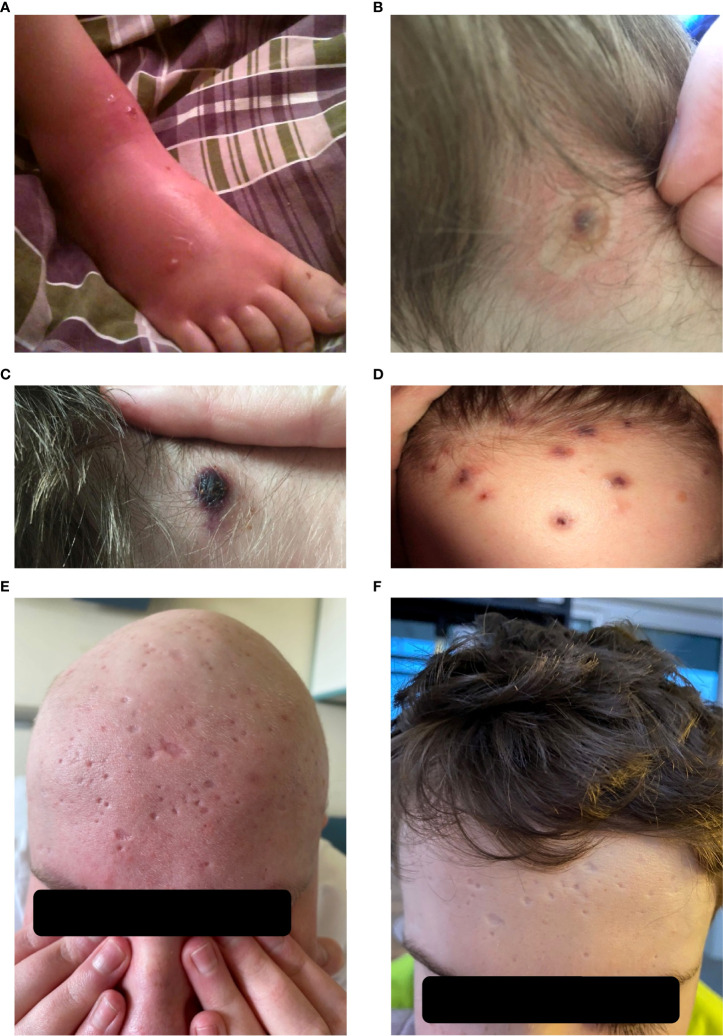
HV lesions.**(A–F)**, **(A)** Insect bite hypersensitivity, **(B)** Vesicles on the neck, **(C)** Bulla and healing erosions, **(D)** Papulopustules on the forehead, **(E)** Typical round punched out varioliform scarring after healing of HV lesions on the forehead before allo-HCT, **(F)** Typical round punched out varioliform scarring after healing of HV lesions on the forehead after allo-HCT.

A 14-year-old boy without burdened family, pregnancy and perinatal history. At 3 months of age, he was diagnosed with cytomegalovirus (CMV) and EBV infection with severe hepatitis, which was confirmed by PCR. The boy did not meet diagnostic criteria for HLH. The parameters of humoral and cellular immunity assessed at that time were normal. At the age of 5, he had an episode of diarrhea, followed by pneumonia complicated by a pleural empyema, which required decortication of the left lung. The patient was also highly hypersensitive to insect bites [**Figure 1A**]. In repeated immunology tests at the age of 5, agammaglobulinemia was observed (IgG 0.5 g/l; IgA <0.06 g/l; IgM 0.12 g/l), the percentage and absolute number of CD19+ cells were normal for the primary lymphocyte subpopulations, the CD3+CD4+/CD3+CD8+ ratio was inverted. In our case NK studies were not performed. In addition, a decreased percentage of memory switched B cells was demonstrated. Genetic tests using the next-generation sequencing method (NGS) were carried out at the age of 13. A mutation in the TNFRSF13B gene was detected, which resulted in defective production of the TACI protein. Thus, the diagnosis of the heterozygous variant of CVID was confirmed and a heterozygous variant in the STX11 gene was also demonstrated.

At the age of 5, substitution therapy with intravenous human immunoglobulin was implemented. After one year, subcutaneous infusions were introduced and carried out for 7 consecutive years, without complications. EBV and CMV viremia was not determined at that time.

**Figure 2:**
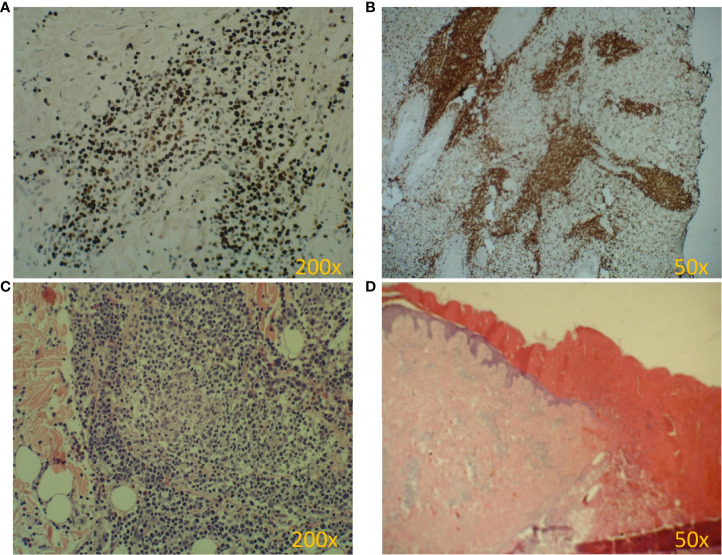
Pathology of HV biopsy specimens. **(A-D)**. **(A)** EBV antigen (EBER) is present in almost every cell. **(B)** Dominant cytotoxic T lymphocytes (CD8+) in the infiltrate. **(C)** Lymphocytic infiltrate in the dermis (H&E). **(D)** Skin ulceration (H&E).

**Figure 3:**
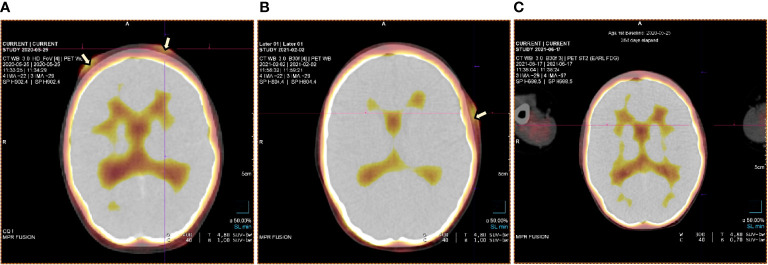
PET test at staging and check-ups at various stages of the therapy **(A-C)**. **(A)** PET test at the time of staging – lesions on the forehead involving the skin and subcutaneous tissue. **(B)** Primary lesions on the forehead subsided. A new leasion occured on the temple - february 2021. **(C)** Complete regression of lesions – june 2021.

**Figure 4:**
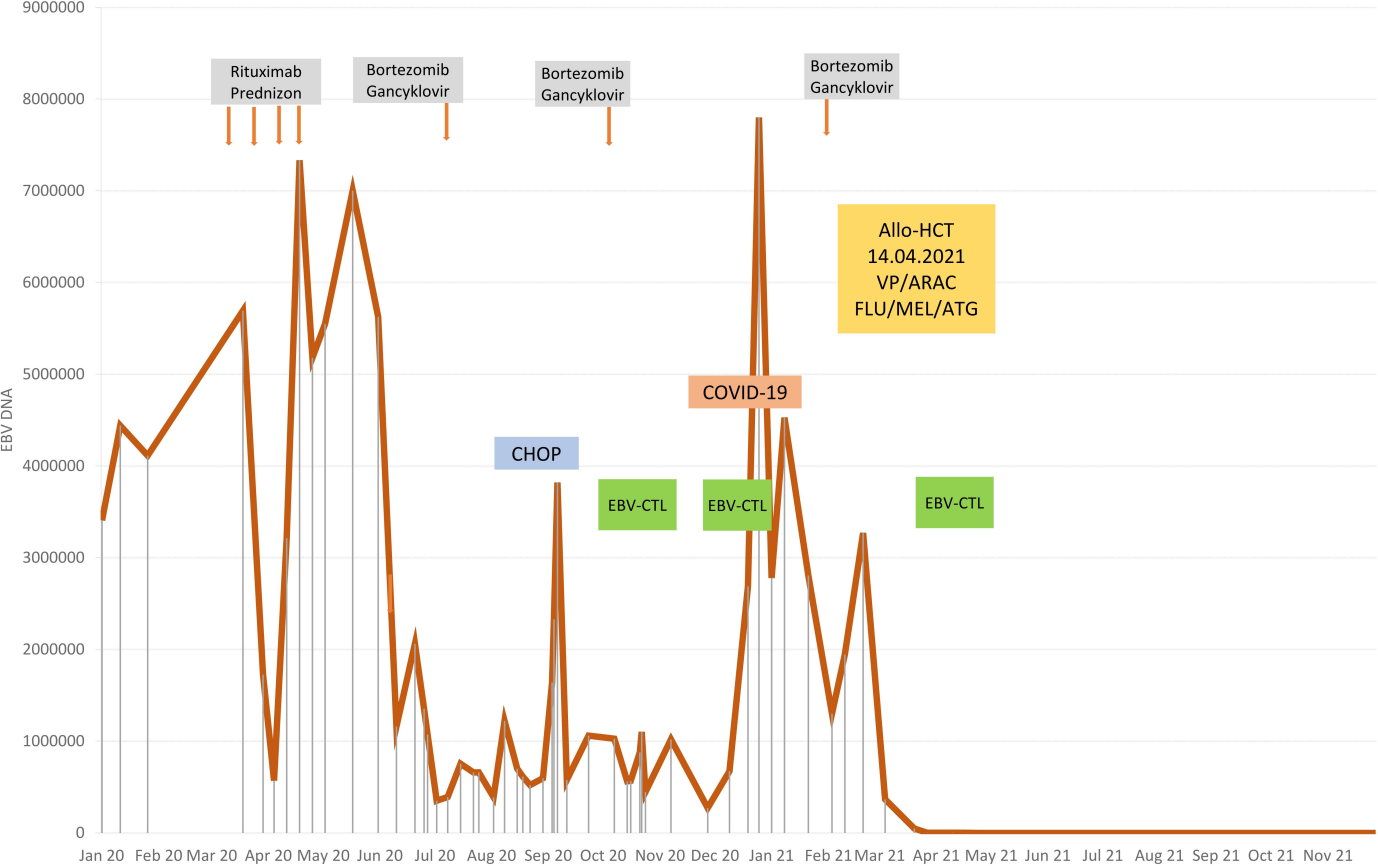
Therapy stages depending on EBV viremia.

At the age of 13, an erythematous papular rash occurred on lower limbs and then papulopustular skin lesions were observed, which receded leaving deep scars. Moreover, periorbital edema was also periodically observed. A relapse of new skin lesions was accompanied by high fever. Laboratory tests revealed thrombocytopenia, neutropenia and hypertransaminasemia. After three months, serous-filled vesicles began to appear around the papular erythematous changes [**Figures 1B, C**]. The skin lesions occurred with periods of exacerbation and remission [**Figure 1D**]. HHV6, Adenovirus, Parvovirus B 19, VZV, HSV 1, HSV 2, HIV, Aspergillus spp, influenza A, influenza B, RSV, SARS-Cov-2, as well as tuberculosis and other mycobacteriosis were excluded. Staphylococcus epidermidis was cultured from swabs of the vesicles on the scalp. Targeted treatment was applied but with no improvement. At the same time, a rapid increase in EBV viremia (>5X106 IU/ml) was observed. In therapy, 4 doses of anti-CD 20 monoclonal antibodies (rituximab) were used. Rituximab was used before the final diagnosis of T-cell involvement was obtained. A transient reduction in EBV viremia was achieved, but with no clinical improvement. The treatment was followed by a complete depletion of B cells. Additionally, a significantly increased percentage and absolute number of CD3+CD8+ cells (cytotoxic T cells) were noticed. A skin biopsy was performed, and histopathological examination showed features of vasculitis. Therefore, prednisone 1 mg/kg body weight was used in the treatment, however with no clinical improvement. Due to the above, histopathological examination of skin tissue samples was performed in a reference center. The consulting pathologist recognized EBER+ CD8. The images of the histopathology results and their description are presented in **Figure 2** [**Figure 2**]. The child was assessed for T-cell receptor (TCR) clonality in peripheral blood T lymphocytes. The result was normal. PET-CT scanning showed features of scalp involvement, with no changes in the lymph nodes, liver and spleen [**Figure 3**]. Based on the above clinical presentation and hystopatologic findings the systemic form of HV-LPD from mature cytotoxic T cells infected with EBV was diagnosed. According to the WHO classification fulfilled criteria were presence of general symptoms, hypersensitivity to insect bites [**Figure 1A**] and no signs of organ involvement (besides skin). The literature analysis showed that the best treatment effects of systemic forms of HV-LPD were achieved in patients in whom the EBV load in peripheral blood was reduced to <200 IU/ml, and then allogeneic hematopoietic cells were transplanted (9). Based on individual reports, different therapeutic regimens were used in the treatment preparing for HCT, as shown in **Figure 4**. None of them resulted in the expected decrease in viral load. Additionally, the disqualification of an unrelated donor made it necessary to postpone the planned HCT for another month. For this reason, it was decided to conduct an experimental cell therapy with the use of T cells sensitized by EBV. Before HCT, the boy underwent 2 cycles of immunotherapy with Tabelecleucel (TabCel) (*i.v.* 2x10^6^/Kg, partially HLA matched) by Atara Biotherapeutics Inc. (Thousand Oaks, CA, USA). TabCel was administered three times in each cycle: on days 1, 8 and 15. The patient tolerated the treatment well, fever appeared on the 6th day after the first administration of the drug. Massive inflammation occurred on the scalp (erythema with subcutaneous swelling, petechiae and dark scabs), which disappeared after one day. Papular eruptions healed quickly with no tendency to deep scar [**Figures 1E, F**]. These changes were treated as the effect of immunotherapy. No such reactions were observed after subsequent intravenous administrations of the TabCel. Periodically, new HV skin lesions appeared that healed quickly leaving scars, but the general symptoms (fever, swelling) subsided. The EBV viremia did not decrease to the expected value of <200 IU/ml. In the first cycle of immunotherapy, the patient was infected with SARS-CoV-2, which was associated with a short-term fever, cough and increased fatigue that lasted for about 4 weeks. Pneumonia with the involvement of about 10% of the lung parenchyma was diagnosed. The treatment included remdesivir, convalescent plasma and empirical antibiotic therapy, which resulted in stabilization of lung lesions. Complications of COVID-19 were mild myocarditis and arterial hypertension. It was necessary to implement dual antihypertensive therapy (enarenal and metoprolol). COVID-19 and its treatment did not interfere with the implementation of specific immunotherapy. In the 8th week after the end of the 2nd cycle of HV-LPD therapy, conditioning according to Sawada (9) was started and then allogeneic hematopoietic cell transplantation was performed from a 10/10 HLA matched unrelated donor, ABO and Rh incompatible with the recipient (5). Cyclosporine and methotrexate were used to prevent graft-versus-host disease (GvHD). On the +5 th day after allo-HCT, a single dose of anti-CD20 antibodies was administered. Due to the positive CMV infection status in the recipient and negative in the donor (high risk of CMV reactivation), letermovir was administered prophylactically. Engraftment was achieved on day +24. The early post-transplant period was complicated by gastrointestinal mucositis. No new skin lesions were formed since HCT. In addition, CMV infection was not reactivated and there were no reports of other infections or fever. Since the administration of the conditioning regimen, a gradual decrease in EBV viremia was observed until undetectable levels on day +17. Due to viremia (10^3^ IU/ml) on day +24, it was decided to administer monoclonal anti-CD20 antibodies single dose as pre-emptive therapy. Symptoms of GvHD were not observed. From day +31, 2 consecutive cycles of EBV immunotherapy with specific cytotoxic T cells were administered on days 1, 8 and 15 of each cycle. Immunosuppressive treatment was completed on day + 134 after allo-HCT.

From day +38 to day +300, no reactivation of EBV and CMV was observed. At the time of preparing the paper (approx. +300 after HCT) the patient’s condition was good. No clinical signs of HV-LPD recurrence occured but there still were elevated levels of transaminases and gamma-glutamyl transferase (GGT). Hematological parameters were normal, except for a slightly reduced number of platelets (in the range of 70-150 K/µl). The boy required systematic human immunoglobulin supplementation, which was carried out from day +180 by the subcutaneous route. In cyclic determinations of immunoglobulin levels around day +280, an increase in IgM concentration was observed, which may be an indication of reconstruction of humoral immunity. There was also an improvement in the analyzed lymphocyte subpopulations. The percentage of CD19+ cells decreased (3.4%), but their absolute number increased to normal (224/µl).

## Discussion

HV-LPD most often affects the population living in Asia and South America (17, 22). Since this disorder is extremely rare in the European population, it can be assumed that in Europeans HV-LPD occurs as a complication of immunodeficiency (2). Infections in CVID are most often of bacterial etiology. Serious viral infections, including CMV and EBV, are less characteristic (8, 24). It cannot be ruled out that in the presented patient the cause of the severe course of EBV and CMV infection in infancy may be a genetic predisposition resulting from the coexistence of STX11 and TACI mutations. The patient did not develop full-blown HLH syndrome, the occurrence of which (in congenital form) requires mutations in both alleles of the STX11 gene. However, perhaps in a patient with a congenital defect in the humoral response, a mutation in allele 1 of the STX11 gene is enough to make the course of acute EBV infection more severe and then to develop into CAEBV. No similar observations were found in the available literature, therefore further research is needed to support this hypothesis. The patient’s case shows that early genetic diagnosis and systematic molecular monitoring of EBV infections in patients with primary humoral deficiency are justified, as they allow for rapid implementation of pre-emptive therapy against CAEBV or EBV-LPD. The genetic tests are basis of EBV diagnostics (25). Only the TACI mutation resulting in the diagnosis of CVID was confirmed in our patient. This mutation is not associated with CAEBV and HV-LPD. The presented case confirmed that skin biopsy plays an important role in the diagnosis of atypical skin lesions in patients with IEI. It is essential that an experienced pathologist performs the histopathological assessment. Due to the rarity of skin lesions typical for HV-LPD, many pathologists will not consider a diagnosis of viral origin. It was similar in the presented case − the first pathologist failed to specify the final diagnosis. Due to the clinical and histopathological similarities of HV and HV-LPD, it is also important to analyze the clonality of the gamma T cell receptor in the section of affected skin (4). Such analysis was not performed in the presented patient. Only the clonality of the TCR VbCD3+ and TCR VbCD3+ CD4+ receptor was assessed demonstrating its polyclonality which, however, does not exclude the diagnosis of HV-LPD.

No standard treatment of HV-LPD has been established so far (4, 10, 17, 21, 22). Previously, thalidomide and chloroquine were used, and now the treatment includes steroid therapy, cyclosporin A, interferon alfa and chemotherapy (4, 10, 17, 21, 22). The treatment of our patient was personalized and adequate both to the disease development and the results of medical examinations. The application of anti-CD20 antibodies is usually effective in the treatment of CAEBV, however, it turned out to have no effect on the patient. In the case of CAEBV-infected B cells, their destruction effectively reduces the EBV load and brings clinical improvement (6). In HV-LPD other cells become infected – these were CD3+ CD8+ T cells in our patient. Therefore, it is not surprising that there was no therapeutic effect after the administration of monoclonal antibodies directed against B cells. Drugs that act on T cells are required for the treatment of HV-LPD. Based on the treatment regimen of T cell lymphomas, an attempt was made to administer cyclophosphamide, doxorubicin, vincristine and prednisone (CHOP) chemotherapy, but with no effect. A similar decision was made by Bollard et al. in a patient with CAEBV (5, 9). Other authors also reported low, only 30% partial effectiveness of chemotherapy in HV-LPD treatment (21). Conventional chemotherapy also turned out to be ineffective in the progression of HV-LPD to systemic lymphoma, hence, there were attempts to implement immunosuppressive therapy in combination with bortezomib and ganciclovir as a bridge to hematopoietic cell transplantation (5, 9, 23, 26). There was an attempt to implement a similar treatment in the presented patient. Steroids, bortezomib and ganciclovir slightly and temporarily reduced the EBV viremia, eliminated general symptoms, but did not cause regression of skin lesions. For the above reasons, other options for optimal preparation of the patient for the HCT procedure were considered. Guided by the experience in the prevention and treatment of PTLD, a decision was made to institute an innovative therapy using EBV-specific allogeneic cytotoxic T cells (tabelecleucel), which resulted in stabilization of the disease, with no evident reduction of EBV viral load, but with virtually no side effects of immunotherapy. The lack of control of EBV viremia may have been caused by long gaps between the EBV-CTL treatment steps. The patient was treated as in PTLD, and it seems that a different strategy should have been implemented, namely: treatment every week until transplantation, and up to approximately 100 days after transplantation, until complete immune reconstruction occurred, causing the immune system to cope with possible reactivation of EBV.

HCT was the next step in the therapy. Hematopoietic cell transplantation (HCT) performed before the disease progression in the stage of irreversible organ damage is considered the most effective method of treatment (5). There are no official recommendations regarding the conditioning for HV-LPD. The diagnosis of refractory CAEBV HV-PLD was an indication for allo-HCT. However, the presence of these presentation of EBV infection in a patient with congenital IEI error was an additional argument in favor of a transplant. In the described case, based on scant literature available, it included early administration of low-dose rabbit antithymocyte globulin (to reduce recipient T cell immunity and enforce donor cell engraftment as well as to decrease the number of EBV-infected T/NK cells for better disease control (9, 10)) and reduced intensity conditioning with thiotepa, cyclophosphamide and fludarabine (20). The use of HCT in the patient confirmed the effectiveness of this therapy. Shortly after HCT, EBV viral load was undetectable and HV-LPD skin lesions subsided. In the post-transplant period, the patient received 2 more TabCell cycles, the assumption was to strengthen anti-EBV effect of transplantation, to reduce the risk of reactivation and further reduction of clinical symptoms of EBV infection. This is an extremely important aspect of treatment as increasing EBV viremia is a risk factor for early relapse and poor prognosis in patients with HV-LPD (19).

## Conclusions

Molecular monitoring is important especially in IEI with T-cell defect, but testing of patients with humoral defects should also be considered.

In case of unclear skin lesions and/or symptoms of lymphoproliferation, it is imperative to consider viral infection as the cause.

Histopathological examination that involves viral pathogens, is an indispensable tool in the diagnostics of HV-LPD in patients with IEI. The preparations should be assessed by an experienced pathologist.

Innovative specific immunotherapy of EBV-CTL (tabelecleucel), used at various stages of treatment, and allo-HCT might be a curative option for patients with HV-LPD in the course of CVID.

Genetic and molecular tests allow for quick and accurate diagnosis, which is of great importance in patients with IEI to avoid complications. The use of combined therapy with tabelecleucel, followed by allo-HCT from an EBV-seropositive donor, allows for clinical and laboratory improvement.

## Patient perspective

The patient is currently 11 months after transplantation of peripheral blood hematopoietic cells from a 10/10 HLA matched unrelated male donor, with ABO and RH incompatibility. The boy remains in complete remission. There was no recurrence of EBV or CMV infection. Complete hematological reconstruction was obtained. Subcutaneous infusions of immunoglobulins are used due to hypogammaglobulinemia. Anti-infective prophylaxis in accordance with guidelines for transplant patients was recommended.

The greatest doubt in the near term remains the fact that scleroderma cGvHD appeared 280 days after HCT, but regressed spectacularly after treatment with steroids, methotrexate and two extracorporeal photopheresis (ECP) treatments.

Late grafts are rare. It is worth analyzing whether cGvHD results from the previously used immunotherapy or whether it is the result of the interaction of two foreign immune systems - the donor and the recipient? Or maybe both of the therapies used?

## Data availability statement

The original contributions presented in the study are included in the article/supplementary material. Further inquiries can be directed to the corresponding author.

## Ethics statement

Written informed consent was obtained from the minor(s)’ legal guardian/next of kin for the publication of any potentially identifiable images or data included in this article.

## Author contributions

All authors listed have made a substantial, direct and intellectual contribution to the work, and approved it for publication.

## Funding

Manuscript financed by the Department of Pediatrics,Hematology and Oncology, Collegium Medicum in Bydgoszcz, Nicolaus Copernicus University in Torun, Poland.

## Conflict of interest

The authors declare that the research was conducted in the absence of any commercial or financial relationships that could be construed as a potential conflict of interest.

## Publisher’s note

All claims expressed in this article are solely those of the authors and do not necessarily represent those of their affiliated organizations, or those of the publisher, the editors and the reviewers. Any product that may be evaluated in this article, or claim that may be made by its manufacturer, is not guaranteed or endorsed by the publisher.
